# Effects of Ultraviolet-C Radiation Exposure on Aircraft Cabin Materials

**DOI:** 10.6028/jres.126.019

**Published:** 2021-08-20

**Authors:** Stephen F. Yates, Giorgio Isella, Emir Rahislic, Spencer Barbour, Lillian Tiznado

**Affiliations:** 1 Honeywell Aerospace, 1300 W. Warner Road, Tempe, AZ 85285, USA; 2 Honeywell Performance Materials and Technology, 15801 Woods Edge Road, Colonial Heights, VA 23834, USA

**Keywords:** aircraft, material compatibility, ultraviolet-C

## Abstract

Ultraviolet-C (UV-C) radiation exposure is an attractive option for rapid and consistent disinfection of interior surfaces in aircraft cabins. In this study, fabric and plastic materials commonly used in aircraft cabins were exposed to UV-C radiation to determine their sensitivity to cumulative damage from frequent application. No significant effect on flame retardancy occurred up to 269 J/cm2 dose, and no effect on tensile or tear strength occurred up to 191 J/cm2 . Changes in color or appearance can occur at lower doses. A limit of 40 J/cm2 is proposed to avoid perceptible changes in appearance.

## 1 Introduction

The coronavirus disease 2019 (COVID-19) pandemic has had a devastating effect on air travel worldwide. In April 2020, the number of passengers on commercial airlines was less than 10% of the number for the previous year, and even in September 2020, the number had only recovered to 35% [1]. Potential air travelers are afraid that they will become infected during the flight, either from other passengers or from virus particles remaining on aircraft cabin surfaces from prior flights. Among other measures, this has prompted airlines to enhance the cleaning done between flights or in the event that an individual becomes ill during a flight. Cleaning methods have included wiping seats, tray tables, or other touch surfaces with chemicals or applying ultraviolet (UV) radiation. For chemical disinfectants, the efficacy of these methods depends on the diligence of the cleaning staff to apply the disinfectants to every surface, and the characteristics of the disinfectants [2]. Similarly, the efficacy of UV radiation depends on the application of a sufficient dose of UV radiation to each surface. Since airline schedules are tight, staff members are under time pressure to complete the cleaning without delaying the flight, which prompts concern that they will not be sufficiently thorough.

Repeated cleaning by either chemical disinfectants or UV radiation may also affect materials in the aircraft cabin. Aircraft cabins typically contain fabrics or leather for the seat coverings, polyester or nylon seat belts, and plastic or metal surfaces. The U.S. Federal Aviation Administration (FAA) has standards for fire retardancy [3], seat belt strength, and other considerations. Care is needed, for chemical disinfectants, to select chemicals and methods to prevent premature damage to these surfaces, and recommendations for their selection and use have been published. Similarly, UV radiation has the potential to damage aircraft cabin materials. For heating, ventilation, and air-conditioning (HVAC) applications, where use of UV radiation is better established, studies have shown that there is dose-dependent damage to plastic materials, including cracking and discoloration [4]. This study explored the dose required to cause damage to materials typically found in commercial aircraft cabins.

Use of germicidal UV radiation is well established for both air treatment and treatment of surfaces [5]. Most commonly, ultraviolet-C (UV-C) light is used, comprising light within the wavelength range of 200 nm to 280 nm. Mercury vapor lamps, with a wavelength of 254 nm, are very commonly used. The germicidal efficiency is a function of wavelength and dose or fluence, which is the intensity of the UV-C radiation at the surface multiplied by the duration of the exposure [6]. The relationship between dose and the germicidal efficiency is shown in Eq. (1), where *H*_0_ is the dose, *D* is the fraction of microorganisms killed, and *K* is a rate constant characteristic of the specific microorganism and the wavelength. Malayeri *et al*. [7] provided a comprehensive collection of the sensitivity of a wide range of microorganisms to UV-C light. For severe acute respiratory syndrome coronavirus (SARS-CoV), two reported measurements of the required dose are available based on clinical studies. Griffiths [8] recently reported that a dose of 5 mJ/cm^2^ resulted in 99% reduction in 6 s in clinical studies. Walker and Ko [9] used a similar coronavirus and reported a constant *K* value of 37.7 × 10^4^ cm^2^/μJ, corresponding, via Eq. (1), to 1.2 mJ/cm^2^ for 99% reduction in clinical studies.

$H_{0}=\;\frac{-ln\left(1-D\right)}{K}\;(1)$

**Fig. 1:**
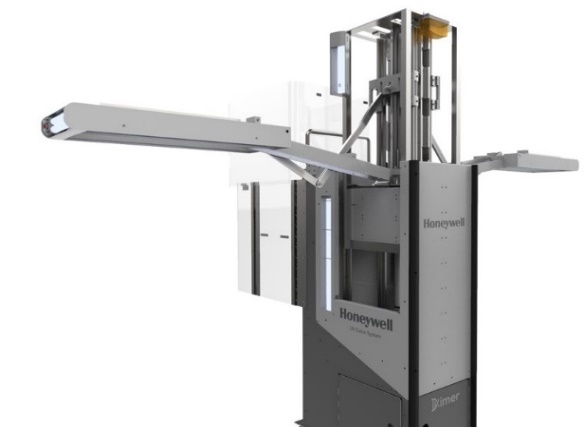
Honeywell UV Cabin System.

Honeywell^1^1 Certain commercial instruments and materials are identified to specify the experimental study adequately. This does not imply recommendation or endorsement by the National Institute of Standards and Technology, nor does it imply that the instruments and materials identified are necessarily the best available for the purpose. has introduced a UV Cabin System (Fig. 1), which is composed of a base that is similar in dimensions to the beverage cart commonly used on aircraft and wings that are deployed to extend over the seats and are equipped with UV-C lamps.^2^2 No testing has been done specifically on inactivation of COVID-19. The system is based on the “Germ Falcon” originally invented by Kreitenberg [10]. This system constitutes a significant improvement over handheld UV-C devices, since it reproducibly applies a known dose of light to each surface, determined by the intensity of the lamps, the distance between the lamps and the surfaces, and the exposure time. The latter is determined by the speed with which the operator moves the device down the aisle of the aircraft, measured by a speedometer on the device. The purpose of this study was to measure the dose incident on aircraft cabin surfaces from the UV Cabin System, and to determine the cumulative effect on aircraft materials of applying this dose.

The intensity of UV-C radiation incident on a surface depends not only on the distance between the lamp and the surface, but also on the angle between the surface and the incident beam. Only surfaces that have direct line of sight to the light source are exposed to UV radiation, unless a UV-reflective surface is present. The effect of shadowing on effectiveness has been described in Refs. [11, 12]. The UV Cabin System mitigates the effect of shadowing relative to a static unit, since it is moved past the surfaces to be treated, changing the angle of UV-C incidence. Nonetheless, surfaces under the seat or inside the overhead luggage compartment are not irradiated.

## 2 Methods

### 2.1 UV Dose Measurements in Simulated Aircraft Cabin

**Fig. 2:**
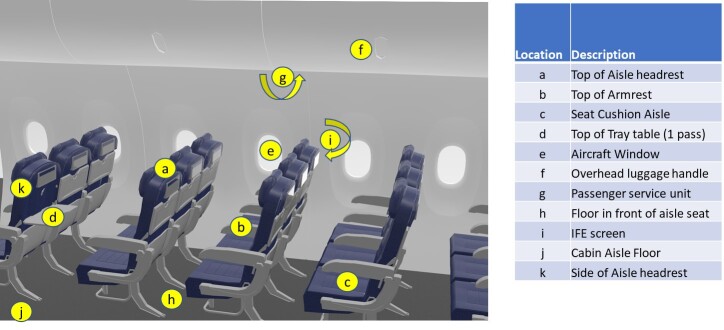
Positions in aircraft cabin selected for dose measurements.

A simulated aircraft cabin was constructed using simple materials, in which the heights and distances of surfaces corresponded to a typical aircraft. A UV-C dosimeter (CureRight Radiometer, Model ILT800, International Light Technologies) was placed in positions and at angles corresponding to aircraft seats, seat backs, floor, windows, overhead compartments, *etc*., as shown in Fig. 2. The UV Cabin System was moved past each surface at a rate of 10 rows per minute (0.17 m/s), and the cumulative single-pass dose was recorded. Table 1 shows these results. Each value is the average of three measurements. Table 1 also shows the number of treatments that would be required to achieve selected dose levels, based on the measured values.

### 2.2 Materials Compatibility Studies

**Fig. 3:**
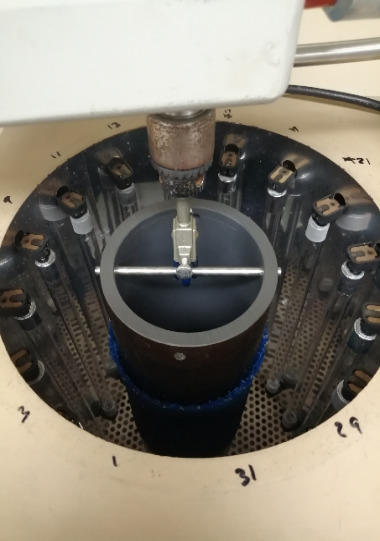
UV irradiation system.

Samples of aircraft materials, including carpet, seat coverings, seat belts, decorative foil laminates, and plastics used for tray tables, *etc*., were obtained from commercial suppliers or airlines. Table 2 shows the materials selected for this study. These samples were exposed to UV-C radiation using two Rayonet reactors (Southern New England Ultraviolet Co., Model RPR-100) equipped with 16 mercury vapor lamps arranged in a cylindrical space (Fig. 3). For flat or flexible materials, samples were taped to the exterior of a short length of polyvinyl chloride (PVC) pipe, which was positioned in the center of the reactor and slowly rotated during the length of exposure. The exposure time was determined by a Tork 457Z timer, which controlled the power to the reactor. A fan in the base of the reactor controlled the temperature to <30 °C. The liquid crystal display (LCD) screen samples were positioned on the base of the reactor at a distance from the UV lamps similar to that for the pipe. The intensity incident on the surface of the PVC pipe was measured by positioning our UV dosimeter (CureRight Radiometer, Model ILT800, International Light Technologies, in intensity mode) at the same distance from the lamps and recording the intensity facing each lamp and facing between each lamp and the next adjacent lamp. These values were averaged.

**Table 1:** Measured treatment doses (average of three replicates) in aircraft and number of treatments corresponding to cumulative doses used in progressive color studies.

**Location**	**Description**	**Single Treatment Dose (mJ/cm^2^) 30 rows/min**	**Number of treatments to reach cumulative dose**			**Years of use at one treatment per day**		
			17 J/cm^2^ dose	34 J/cm^2^ dose	51 J/cm^2^ dose	17 J/cm^2^ dose	e	51 J/cm^2^ dose
**a**	Top of aisle headrest	13.0 ± 0.4	1311	2622	3933	3.6	7.2	10.8
**b**	Top of armrest	9.4 ± 0.1	1806	3613	5419	4.9	9.9	14.8
**c**	Aisle seat cushion	7.1 ± 0.2	2387	4774	7161	6.5	13.1	19.6
**d**	Top of tray table (one pass)	5.3 ± 0.1	3235	6469	9704	8.9	17.7	26.6
**e**	Aircraft window	3.4 ± 0.1	5055	10,109	15,164	13.8	27.7	41.5
**f**	Overhead luggage handle	3.5 ± 0.2	4902	9805	14,707	13.4	26.9	40.3
**g**	Passenger service unit	3.2 ± 0.4	5238	10,476	15,714	14.4	28.7	43.1
**h**	Floor in front of aisle seat	6.1 ± 0.1	2777	5554	8330	7.6	15.2	22.8
**i**	In-flight entertainment screen	7.0 ± 0.1	2429	4857	7286	6.7	13.3	20.0
**j**	Aisle cabin floor	5.1 ± 0.4	3305	6609	9914	9.1	18.1	27.2
**k**	Side of aisle headrest	9.1 ± 0.1	1861	3723	5584	5.1	10.2	15.3

**Table 2:** Selected materials for materials compatibility study.

**Material Identification**	**Material Type**	**Description/Use**	**Vendor**
**A**	Sateen Leather, Moon Gray LL-3442	Natural leather, used for premium seat and pane coverings	Douglass Interior Products
**B**	Columbia Synthetic Leather Glacier DEF-CD287	Seat covering, midrange aircrafts	
**C**	Luxaire Synthetic Leather Nickel CD47-AR175FR	Most popular synthetic leather for midrange aircraft used for seat covering	
**D**	Synthetic Leather 590-5012FR12 Zephyr	Seat covering	Ultrafabrics, Inc.
**E**	Synthetic Leather 492-6022FR12 Hydra	Seat covering	
**F**	Heavy Duty Wool-Polyester Blend DEF-7284/0045	Economy seat cover of choice for European Union carriers, primary wool blend	Douglass Interior Products
**E**	Heavy Duty Wool-Polyester Blend DEF-7898/48	Economy seat cover of choice for U.S. carriers, primary polyester blend	
**H**	Polyester Seat Belt Webbing	Aircraft seat belt	Aircraft Belts, Inc.
**I**	Boltaron 9815N	Thermoformed aircraft interior parts	SIMONA Boltaron Inc.
**J**	Kydex Polyacrylate 7200ST	Tray tables, side paneling, seat paneling, overhead bins	Sekisui Kydex
**E**	ProLens Aircraft Grade Polycarbonate	Window dust cover, lenses, shades, class dividers	Professional Plastics
**L**	Decorative Foil Laminate S3863	Aircraft interior decorative laminate	Schneller Aircraft Interior Designs
**M**	Decorative Foil Laminate S016329	Aircraft interior decorative laminate	
**N**	Decorative Foil Laminate S05051-011-H5	Aircraft interior decorative laminate	
**O**	Decorative Foil Laminate S12335	Aircraft interior decorative laminate	
**P**	Nylon Carpet Humility First AB-7400/7664	Carpet	Douglass Interior Products
**Q**	Ovation Select Arm-mount 9″ Monitor 990-E0890-045	In-flight entertainment screen	Honeywell
**R**	10.1" Touchscreen 990-E1010-001	In-flight entertainment screen	

Measurements for flame retardancy were made by AeroBlaze, Inc., using the method prescribed by the FAA [3]. Tensile strength and tear strength for the material samples were measured at Honeywell’s internal laboratory using test methods ASTM D5035-11 [11], ASTM D5587-15 [12], and ASTM D2261-17 [13]. The tensile strength for the seat belt samples was measured using SAE International Standard AS8043 [14], and tensile strength for polycarbonate sheets was measured using ASTM D638-14, Type 1 [15], by Element Los Angeles. A dynamic mechanical analysis (DMA) study on the polycarbonate sheets was also completed by Element Los Angeles using ASTM method D7028-07 [16]. The effect of UV-C radiation on the appearance of samples was evaluated using a progressive exposure experiment. In this experiment, portions of the sample were masked using masking tape, and the sample was exposed to UV radiation. Then, one section of the tape was removed, and an additional dose of UV radiation was applied. This process was continued to expose the remaining sections, leaving one section masked the entire time as the control. A photograph of the sample was taken.

## 3 Results

The effect of exposure to UV-C radiation on the flame retardancy of materials is reported in Tables 3–5. Seat coverings and samples of plastics were tested using the FAA vertical burn test. This test involves holding an open flame against the bottom of a hanging strip of fabric and measuring the time required for the burning sample to self-extinguish or stop dripping, as well as the length the fire travels up the fabric before it self-extinguishes. To pass the test, the flame time must be less than 15 s, the flame drip time must be less than 5 s, and the burn length must be less than 20 cm (8 in.). Tables 3–5 show the results of this study. Where flame time or drip time are shown as zero, the samples did not ignite or drip. The UV-C doses chosen were higher than the cumulative doses shown in Table 1 and represent extreme values, which could occur after many years of repetitive use. In all cases, the measured values met the specified limit for the FAA standard.

Some plastics must also pass the same test. Those tested in this way included Boltaron 9815N, commonly used for tray tables and seat housings, and ProLens polycarbonate, used for the transparent dust covers for windows. The FAA flame retardancy requirements are the same for these as for the fabrics, and they also passed the test, even after prolonged UV irradiation.

As prescribed by FAA regulations [3], our seat belt and carpet samples were tested for flame retardancy using the horizontal burn method instead of the vertical method. The results are shown in Table 6. Using this test, the requirement is that the burn rate be less than 6.4 or 10.2 cm/min (2.5 or 4 in./min). As the results in the table show, UV-C radiation had little effect on flame retardancy, and the samples in all cases passed this test.

The effect of exposure of UV-C radiation on fabrics used for seat coverings or for the carpet was measured by comparing the tensile strength (both in the machine and cross-machine directions) and trapezoid tear strength as a function of UV-C exposure. For these measurements, no standard was identified, so our criterion was that no consistent change in strength greater than one standard deviation would be measured. Table 7 shows the tensile strength results, and Table 8 shows the trapezoid tear strength results. Because Sample C, the Luxaire synthetic leather, had a knit backing, and knits are frequently highly extensible, we chose to use the tongue tear method for this sample (Table 9). As the tables show, for all samples, there was no significant change in fabric strength as a result of exposure to UV-C radiation, even though the doses applied were significantly in excess of the doses that would be seen during use.

**Table 3:** Flame time: Vertical burn results {per 14 CFR 25, Appendix F, Part I(b)(4) [3]} for aircraft materials after various levels of UV-C radiation exposure. Number of replicates per condition = 3. Success criterion for flame time average = 15 s maximum.

**Material ID**	**Exposure**	**Flame Time (s)**						
		0 J/cm^2^	27 J/cm^2^	54 J/cm^2^	76 J/cm^2^	134 J/cm^2^	269 J/cm^2^	382 J/cm^2^
**A**	Avg.	0	0	0	—	0	0	—
	Std. Dev.	0	0	0	—	0	0	—
**B**	Avg.	0	0	0	—	1.2	0.8	—
	Std. Dev.	0	0	0	—	0.9	1.1	—
**C**	Avg.	0	0	0	—	0	0	—
	Std. Dev.	0	0	0	—	0	0	—
**E**	Avg.	0	0	0	—	0	0	—
	Std. Dev.	0	0	0	—	0	0	—
**F**	Avg.	0	0	0.7	—	0	0	—
	Std. Dev.	0	0	1.2	—	0	0	—
**G**	Avg.	0	0	0	—	0	0	—
	Std. Dev.	0	0	0	—	0	0	—
**I**	Avg.	0	—	—	0	—	—	0
	Std. Dev.	0	—	—	0	—	—	0
**K**	Avg.	2.2	—	—	0.5	—	—	2.0
	Std. Dev.	1.3	—	—	0.9	—	—	0.3

**Table 4:** Drip time: Vertical burn results {per 14 CFR 25, Appendix F, Part I(b)(4) [3]} for aircraft materials after various levels of UV-C radiation exposure. Number of replicates per condition = 3. Success criterion for drip flame time average = 5 s maximum.

**Material ID**	**Exposure**	**Drip Time (s)**						
		0 J/cm^2^	27 J/cm^2^	54 J/cm^2^	76 J/cm^2^	134 J/cm^2^	269 J/cm^2^	382 J/cm2
**A**	Avg.	0	0	0	—	0	0	—
	Std. Dev.	0	0	0	—	0	0	—
**B**	Avg.	0	0	0	—	0	0	—
	Std. Dev.	0	0	0	—	0	0	—
**C**	Avg.	0	0	0	—	0	0	—
	Std. Dev.	0	0	0	—	0	0	—
**E**	Avg.	0	0	0	—	0	0	—
	Std. Dev.	0	0	0	—	0	0	—
**F**	Avg.	0	0	0	—	0	0	—
	Std. Dev.	0	0	0	—	0	0	—
**G**	Avg.	0	0	0	—	0	0	—
	Std. Dev.	0	0	0	—	0	0	—
**I**	Avg.	0	—	—	0	—	—	0
	Std. Dev.	0	—	—	0	—	—	0
**K**	Avg.	0	—	—	0	—	—	0
	Std. Dev.	0	—	—	0	—	—	0

**Table 5:** Burn rate: Vertical burn results {per 14 CFR 25, Appendix F, Part I(b)(4) [3]} for aircraft materials after various levels of UV-C radiation exposure. Number of replicates per condition = 3. Success criterion for burn length average = 20.32 cm (8 in.) maximum.

**Material ID**	**Exposure**	**Burn Length (cm [in.])**						
		0 J/cm^2^	27 J/cm^2^	54 J/cm^2^	76 J/cm^2^	134 J/cm^2^	269 J/cm^2^	382 J/cm^2^
**A**	Avg.	3.30 (1.30)	2.46 (0.97)	1.19 (0.47)	—	2.29 (0.90)	1.44 (0.57)	—
	Std. Dev.	0.72 (0.28)	0.53 (0.21)	0.15 (0.06)	—	0.55 (0.90)	0.15 (0.06)	—
**B**	Avg.	5.50 (2.17)	6.43 (2.53)	6.43 (2.53)	—	6.35 (2.50)	5.93 (2.33)	—
	Std. Dev.	0.15 (0.06)	0.15 (0.06)	0.43 (0.17)	—	0	0.15 (0.06)	—
**C**	Avg.	5.67 (2.23)	5.50 (2.17)	5.25 (2.07)	—	4.40 (1.73)	4.15 (1.63)	—
	Std. Dev.	0.15 (0.06)	0.15 (0.06)	0.15 (0.06)	—	0.15 (0.06)	0.15 (0.06)	—
**E**	Avg.	5.50 (2.17)	5.93 (2.33)	6.52 (2.57)	—	6.77 (2.67)	6.52 (2.57)	—
	Std. Dev.	0.15 (0.06)	0.15 (0.06)	0.15 (0.06)	—	0.39 (0.15)	0.15 (0.06)	—
**F**	Avg.	4.57 (1.80)	4.57 (1.80)	4.32 (1.70)	—	3.81 (1.50)	3.81 (1.50)	—
	Std. Dev.	0.5 (0.20)	0	0	—	0	0.3 (0.10)	—
**G**	Avg.	6.52 (2.57)	6.10 (2.40	6.35 (2.50)	—	7.11 (2.80)	8.72 (3.43)	—
	Std. Dev.	0.29 (0.12)	0.44 (0.17)	0.44 (0.17)	—	1.11 (0.44)	0.64 (0.25)	—
**I**	Avg.	0.93 (0.37)	—	—	1.02 (0.40)	—	—	1.02 (0.40)
	Std. Dev.	0.15 (0.06)	—	—	0	—	—	0
**K**	Avg.	1.61 (0.63)	—	—	1.52 (0.60)	—	—	1.27 (0.50)
	Std. Dev.	0.15 (0.06)	—	—	0.25 (0.10)	—	—	0

**Table 6:** Horizontal burn results {per 14 CFR 25, Appendix F, Part I(b)(5) [3]} for aircraft materials after various levels of UV-C radiation exposure. Number of replicates per condition = 3. Success criterion for average burn rate must not exceed 6.4 or 10.2 cm/min (2.5 or 4.0 in./min) depending on the type of material.

**Material ID**	**Exposure**	**Time to Cross 1.5 in. Mark (s)**					**Burn Length (cm [in.])**				
		0 J/cm^2^	27 J/cm^2^	54 J/cm^2^	134 J/cm^2^	269 J/cm^2^	0 J/cm^2^	27 J/cm^2^	54 J/cm^2^	134 J/cm^2^	269 J/cm^2^
**H**	Avg.	0	0	16.4	0	35.2	2.37 (0.93)	1.86 (0.73)	3.05 (1.20)	1.86 (0.73)	2.54 (1.00)
	Std. Dev.	0	0	28.5	0	61	0.15 (0.06)	0.15 (0.06)	2.42 (0.95)	0.39 (0.15)	1.32 (0.52)
**P**	Avg.	155.1	169.9	168.3	218.1	182.3	11.30 (4.45)	10.48 (4.13)	9.65 (3.80)	12.36 (4.87)	9.27 (3.65)
	Std. Dev.	2.8	27.8	19.9	22.7	29.5	0.54 (0.21)	3.13 (1.23)	0.51 (0.20)	1.03 (0.40)	2.73 (1.08)

**Table:** 

**Material ID**	**Exposure**	**Total Time (s)**					**Burn Rate (cm/min [in./min])**				
		0 J/cm^2^	27 J/cm^2^	54 J/cm^2^	134 J/cm^2^	269 J/cm^2^	0 J/cm^2^	27 J/cm^2^	54 J/cm^2^	134 J/cm^2^	269 J/cm^2^
**H**	Avg.	59.7	50.5	65.3	57.1	72.7	2.4 (0.94)	2.2 (0.88)	2.6 (1.01)	2.0 (0.77)	2.2 (0.85)
	Std. Dev.	6.1	3.7	35.2	10	42.1	0.13 (0.05)	0.32 (0.13)	0.65 (0.25)	0.22 (0.08)	0.25 (0.10)
**P**	Avg.	461.3	432.5	410.8	520.1	435	1.47 (0.58)	1.45 (0.57)	1.41 (0.54)	1.42 (0.56)	1.26 (0.50)
	Std. Dev.	2.8	32.7	19.5	22.1	96.3	0.06 (0.02)	0.39 (0.15)	0.04 (0.02)	0.07 (0.03)	0.13 (0.02)

**Table 7:** Tensile strength (ASTM D5035-11) of aircraft materials after various levels of UV-C radiation exposure, where number of replicates per condition = 5.

**Material**	**Exposure**	**Tensile Strength (N) in Machine Direction**			**Tensile Strength (N) in Cross-Machine Direction**		
		0 J/cm^2^	76 J/cm^2^	191 J/cm^2^	0 J/cm^2^	76 J/cm^2^	191 J/cm^2^
**A**	Avg.	273.7	241.9	240.6	N/A	N/A	N/A
	Std. Dev.	23.0	40.4	32.9	N/A	N/A	N/A
**B**	Avg.	276.0	274.7	270.1	285.4	292.8	275.3
	Std. Dev.	8.0	5.1	12.8	14.2	14.8	21.6
**C**	Avg.	309.1	298.8	308.6	167.7	173.2	169.4
	Std. Dev.	9.9	14.8	23.2	8.7	6.6	2.1
**D**	Avg.	793.9	818.7	824.3	355.4	334.3	341.4
	Std. Dev.	51.1	32.4	14.5	19.2	25.3	28.4
**E**	Avg.	782.3	796.8	785.7	392.2	429.1	397.7
	Std. Dev.	46.5	22.4	28.2	31.2	18.5	19.1
**F**	Avg.	396.6	373.7	409.1	392.3	368.9	375.6
	Std. Dev.	23.1	16.3	33.0	25.3	21.3	10.9
**G**	Avg.	954.9	945.0	977.5	722.3	719.3	681.8
	Std. Dev.	43.7	29.8	31.9	21.7	33.8	21.3
**P**	Avg.	748.7	753.4	728.5	457.3	454.1	442.5
	Std. Dev.	20.0	38.7	29.9	23.9	16.0	12.6

**Table 8:** Trapezoid tear strength (ASTM D5587-15) of aircraft materials after various levels of UV-C radiation exposure, where number of replicates per condition = 3.

**Material**	**Exposure**	**Tear Strength (N) in Machine Direction**			**Tear Strength (N) in Cross-Machine Direction**		
		0 J/cm^2^	76 J/cm^2^	191 J/cm^2^	0 J/cm^2^	76 J/cm^2^	191 J/cm^2^
**A**	Avg.	78.4	83.9	79.5	N/A	N/A	N/A
	Std. Dev.	9.0	9.0	11.9	N/A	N/A	N/A
**B**	Avg.	38.8	40.6	41.1	66.3	65.2	66.1
	Std. Dev.	2.4	2.5	2.4	9.0	0.9	1.7
**D**	Avg.	151.2	155.2	151.5	92.1	90.7	92.5
	Std. Dev.	5.4	5.7	7.5	5.5	0.7	5.3
**E**	Avg.	137.0	136.3	140.4	114.8	105.6	107.2
	Std. Dev.	2.5	10.4	6.9	3.8	5.1	4.7
**F**	Avg.	238.3	237.4	231.8	304.2	289.6	304.0
	Std. Dev.	36.0	39.1	16.2	18.4	16.5	32.3
**G**	Avg.	314.6	319.2	309.4	211.4	217.1	213.5
	Std. Dev.	8.4	25.7	23.7	6.7	14.3	4.9
**P**	Avg.	324.0	339.3	339.2	293.4	315.1	291.5
	Std. Dev.	23.9	18.8	27.3	12.4	19.0	25.4

**Table 9:** Tongue tear strength (ASTM D2261-17) of aircraft materials after various levels of UV-C radiation exposure, where number of replicates per condition = 3.

**Material**	**Exposure**	**Tear Strength (N) in Machine Direction**			**Tear Strength (N) in Cross-Machine Direction**		
		0 J/cm^2^	76 J/cm^2^	191 J/cm^2^	0 J/cm^2^	76 J/cm^2^	191 J/cm^2^
**C**	Avg.	38.1	38.8	39.1	27.1	26.8	25.5
	Std. Dev.	1.1	0.9	0.4	1.1	1.1	0.4

Seat belt strength is obviously critical for safety, and an SAE test [14] was conducted by Element Los Angeles using samples exposed to UV-C radiation at various doses (Table 10). Since seat belts may easily be flipped on a seat, these samples were exposed to the indicated dose on each side. The SAE requirement is that the breaking strength for the seat belt exceed 5000 lbf (22.24 kN), and that the percent elongation at 11.12 kN (2500 lbf) be less than 20%. Table 10 shows that there was a small increase in the breaking strength with exposure to UV-C radiation, and that all samples met the SAE criteria.

**Table 10:** Tensile strength for seat belt sample H (SAE AS8043) after various levels of UV-C exposure.

UV Dose (J/cm^2^)	Breaking Strength (kN)	Std. Dev. (kN)	Elongation at 11.12 kN (%)	Std. Dev. (%)
0	27.68	1.30	14.4	1.9
54	31.75	2.08	11.7	
153	31.59	0.87	14.4	1.9
307	31.78	0.07	13.3	0
Requirement	> 22.24		< 20	

The effect of exposure to UV-C radiation on polycarbonates used for window dust coverings, class partitions, and light lenses was measured by comparing the tensile strength and viscoelastic properties as a function of exposure to UV-C radiation. For these measurements, no minimum requirement was identified, so our criterion was that no consistent change in properties greater than one standard deviation be measured. Table 11 shows the tensile strength results, and Table 12 shows the glass transition onset and peak for the storage (E′) and loss (E″) modulus determined from DMA, as well as the glass transition temperature as measured using the tan delta peak temperature. Only two samples from each exposure levels were tested through DMA, so standard deviation is not reported. However, for all levels of exposure, the results were within 1 °C. As the tables show, there was no significant change in properties as a result of exposure to UV-C radiation, even though the doses applied were significantly in excess of what would be seen during use. The effect of exposure to UV-C radiation on transparency of the polycarbonate is discussed below.

**Table 11:** Tensile strength for ProLens aircraft-grade polycarbonate K (ASTM D638-14 [17]) after various levels of UV-C exposure.

UV Dose (J/cm^2^)	Peak Stress (MPa)	Standard Deviation (MPa)	Modulus (MPa)	Standard Deviation (MPa)
0	65.4	0.34	2.54	0.10
76	65.4	0.37	2.43	0.16
382	65.4	0.12	2.49	0.092

**Table 12:** Dynamic mechanical analysis: glass transition temperature for ProLens aircraft-grade polycarbonate K {ASTM D7028-07 (2015) [18]} after various levels of UV-C exposure.

UV Dose (J/cm^2^)	*E*′ Onset (°C)	*E*″ Peak (°C)	Tangent Delta Peak Temperature (°C)
0	148	153	157
76	149	152	156
382	149	153	156

**Fig. 4:**
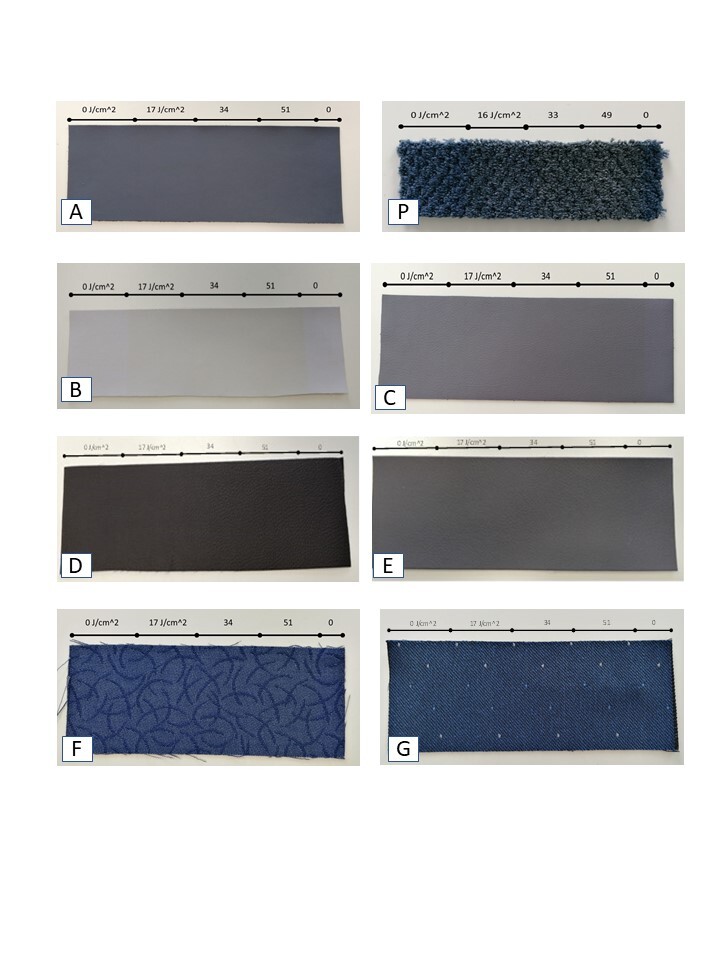
Progressive color study photographs for carpet and seat-covering fabrics.

To evaluate the effect of UV-C on the appearance of materials, a study was completed on each. The materials were partially masked with tape, exposed to UV light, and then progressively unmasked. The effect is to create zones with different UV-C dose within the same sample. The samples were evaluated visually, and photographs of these samples were taken. Figure 4 shows photos of fabrics used for seat coverings and for carpet. Our inspection showed no apparent color changes for the leather seat covering A, or the two wool-polyester fabric coatings F and G. The nylon carpet sample P appeared a bit grayer after UV-C exposure. Of the four synthetic leather samples, two (D and E) had no apparent color change, while C was slightly yellowed at the highest dose. Sample B had a more visible yellow tinge at the highest dose. This may have been due to its lighter color.

**Fig. 5:**
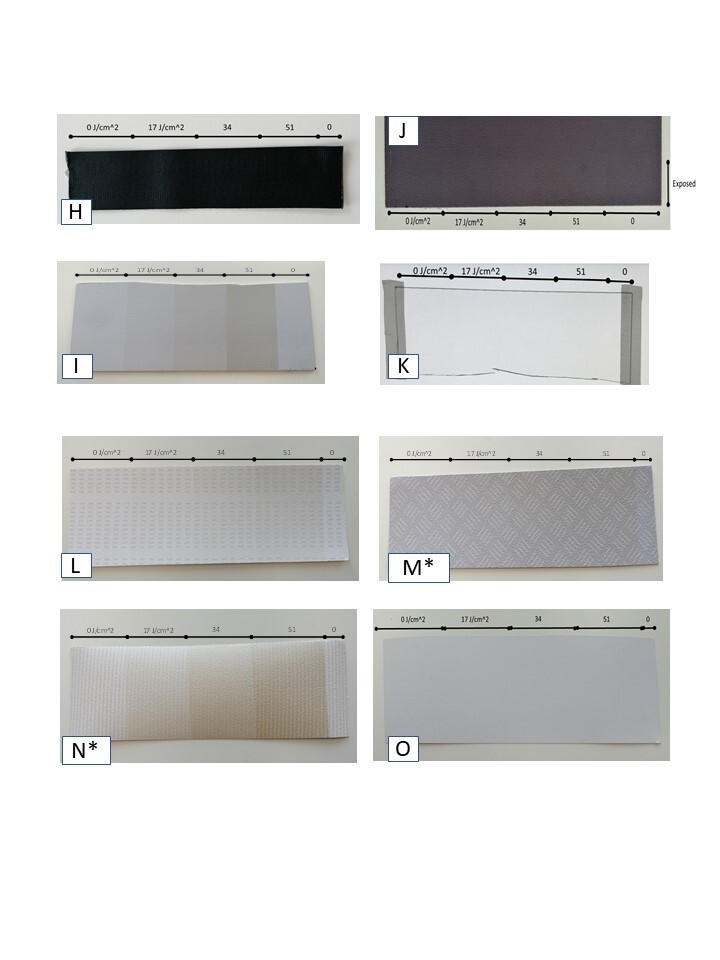
Progressive color study photographs for other materials (seat belt, plastics, and décor foil laminates). Samples with * contained heat-activated adhesive.

Figure 5 shows other materials that were evaluated for changes in color. A black seat belt sample H showed no apparent change in appearance. Samples of plastics commonly used for tray tables, the bezels of in-flight entertainment screens, seat shells, and other uses were tested. A dark-colored sample of Kydex polyacrylate J showed no apparent change in color, but a light-colored sample of Boltaron 9815N was the most sensitive material tested to UV-C exposure, and it progressively yellowed with increasing UV-C exposure. The image of transparent polycarbonate K was photographed taped against a white illuminated screen for better visibility. A faint trace of yellow was seen at the highest dose. Decorative foil laminates are commonly used on aircraft walls or partitions, and four samples were evaluated. Samples L, M, and N showed some yellowing at the highest dose, with N, the most sensitive, showing discoloration at lower doses. Sample O had no apparent color change. On investigation, the two most sensitive samples, M and N, were marked as containing a heat-activated adhesive. In normal use, this adhesive would be used to apply the laminate to an interior surface in the cabin.

**Fig. 6:**
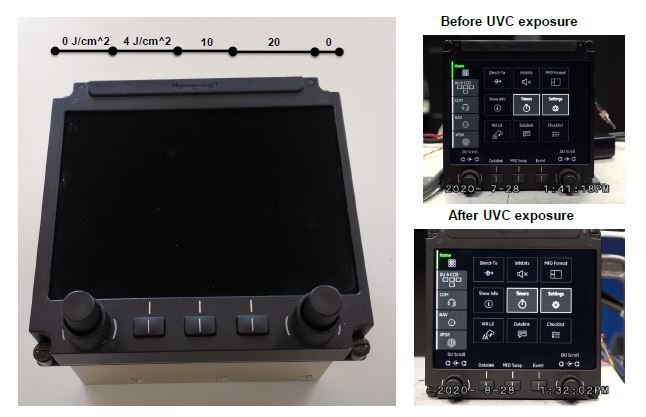
Honeywell TSC 2.0 cockpit touchscreen display. Left: UV progressive doses. Right: Display before and after UV-C exposure.

Increasingly, aircraft cabins are equipped with LCD screens for in-flight entertainment and other uses. In the cabin, these may be mounted on the back of the seat in front of the passenger or on swing-out or fold-out mounts. Similar displays are used in the cockpit. A sample LCD screen in the form of a cockpit display was exposed to UV-C light in the same manner as with the progressive exposure experiments. Prior to this exposure, the monitor was activated, and after the exposure, the same display was again viewed. Figure 6 shows the results of the experiment. No discoloration or fading occurred for the bezel or other plastic components, nor to the screen itself. The screen continued to function, and, as the before and after photos reveal, there was no effect on the appearance of the display.

## 4 Discussion

The results in Tables 3–6 show that, for a variety of materials, there was no significant impact of UV-C exposure on flame retardancy, even for doses much more extreme than would be expected for normal use. The highest dose used in the study, 269 J/cm^2^, is more than 20,000 times the single treatment dose (13 mJ/cm^2^) from Table 1 in the most heavily exposed location (top of the aisle seat headrest). If cleaning was performed at the 30 rows/min pace, once a day, it would require more than 56 years of use to reach this dose. Similarly, there was no significant impact of exposure to UV-C radiation on the tensile or tear strength of the materials in Tables 7–11 at the highest dose tested, 191 J/cm^2^. This is 14,692 times the single treatment dose at the top of the headrest and would correspond to more than 40 years of daily use. These results are not surprising considering that these materials are opaque to UV radiation. As a result, UV radiation could only have an effect on the exposed surfaces, while bulk properties like flame retardancy or strength are unaffected. While the scope of this study focused on isolating the effects of UV-C exposure on aircraft interior materials by exposing and testing them in controlled environments, it is important to consider real-life dynamic and environmental factors that may exacerbate the effects of UV-C on the mechanical performance of the materials. Dynamic in-use factors may include abrasion, mechanical loading, and cyclic fatigue. Environmental factors may include temperature, humidity, and chemical exposure. To determine the compounding effects of these factors with UV-C, further studies are necessary.

The effect of exposure to UV-C radiation on the color or appearance of the materials tested was more significant. Darkly colored fabrics or materials generally did not show much color change, but white or light-colored materials became perceptibly faded or yellowed at the higher dose values. Of the seat fabrics, the wool-polyester blend materials and the leather appeared to be the most resistant to color change, while synthetic leather samples became slightly yellowed. Of the plastic materials, Boltaron 9815N, believed to be a polyacrylate PVC blend, was relatively sensitive to UV exposure, while polycarbonate was only slightly affected. Two of the decorative foil laminates (part numbers S016329 and S05051-011-H5) were marked as containing a heat-activated adhesive. In use, these laminates would be applied to a surface and heated to adhere. These two samples were also more sensitive to exposure to UV-C radiation. Since in practice the laminates will be exposed in place in an aircraft, these two samples are less representative.

Based on the effect on color, an overall cumulative exposure limit of 40 J/cm^2^ represents a UV-C dose that would correspond to at most slight color changes relative to unexposed materials. For seat coverings, using the most highly exposed location at the aisle seat head rest (13 mJ/cm^2^), this cumulative exposure would correspond to 3077 treatments, while for tray tables (5.3 mJ/cm^2^), it would correspond to 7547 treatments, and for wall and windows (3.4 mJ/cm^2^), the corresponding number of treatments would be 11,764. In many cases, airlines would replace seats or other materials for other aesthetic reasons before these limits were reached.

## 5 Conclusions

Airlines need to increase passenger confidence to return to profitability. Cleaning aircraft cabin surfaces plays in important role in restoring confidence, and cleaning options that can be accomplished rapidly and consistently are valuable. Exposure to UV-C radiation has proven efficacy in non-aircraft settings, which makes use of this technology in aircraft cabins attractive. This study has shown that materials in the cabin are unlikely to be damaged by repeated exposures to UV-C radiation. Flame retardancy was unaffected for all tested materials up to 269 J/cm^2^, and strength was unaffected up to 191 J/cm^2^. Exposure to UV-C radiation can affect color and appearance of aircraft cabin interior components, and a useful exposure limit to avoid changes in appearance of 40 J/cm^2^ is proposed. Because UV-C has been proven capable of inactivating various viruses and bacteria when properly applied, has no known adverse safety-related effects, and has no noticeable color or appearance effects on aircraft materials for a significant length of exposure time, airlines may find that UV-C radiation is preferable to the use of disinfectant chemicals.
